# Effect of a Nano-Sized Lipid-Based Eye Drop on Diabetic Dry Eye

**DOI:** 10.3390/biomedicines13040763

**Published:** 2025-03-21

**Authors:** Rosario Gulias-Cañizo, Pablo Alexis Limón-Zurita, Jimena Ceja-Martínez, Oscar Guerrero-Berger

**Affiliations:** 1 Clinical Research, Fundación Hospital Nuestra Señora de la Luz, Mexico City 06030, Mexico; 2 Centro Oftalmológico Mira, Taxco 35, Roma Sur, Cuauhtémoc, Mexico City 03840, Mexico; 3 Department of Anterior Segment Surgery, Fundación Hospital Nuestra Señora de la Luz, Ezequiel Montes 135, Tabacalera, Cuauhtémoc, Mexico City 06030, Mexico

**Keywords:** dry eye, OSDI, ocular surface disease index, non-invasive keratograph break-up time, NIKBUT, NIBUT, K5M, tear break-up time, nanoemulsion, HP-guar

## Abstract

**Background**: Dry eye disease (DED) is currently recognized as a global health concern, with a prevalence that ranges from 5% to 75%. Given the severity of dry eye in diabetic patients and the global prevalence of diabetes, it is crucial to evaluate new treatments that potentially improve tear film stability in this patient population. **Methods**: Single-center, open-label, single-arm, and interventional study in adult patients with type-2 diabetes mellitus and all DED subtypes evaluating a propylene glycol-hydroxypropyl guar nanoemulsion that has shown in previous studies to improve tear film stability in nondiabetic patients. **Results**: After 28 days of treatment, the Ocular Surface Disease Index (OSDI) scores showed significant improvement, decreasing from a baseline mean of 42.72 ± 17.69 to 25.53 ± 17.14 on Day 28 (*p* < 0.001); Non-Invasive Keratograph Break-Up Time (NIKBUT) also improved significantly, increasing from 3.45 ± 1.17 s at baseline to 5.94 ± 1.48 s on Day 28 (*p* < 0.001). No significant changes were observed in the infrared meibography score (baseline: 1.48 ± 0.93 vs. Day 28: 1.47 ± 0.92, *p* = 0.279), tear meniscus height (TMH) (baseline: 0.25 ± 0.10 mm vs. Day 28: 0.25 ± 0.08 mm, *p* = 0.086), or meibomian gland expressibility score (MGES). The redness score significantly decreased from 1.88 ± 0.68 at baseline to 1.40 ± 0.59 on Day 28 (*p* < 0.001). **Conclusions**: These findings suggest notable improvements in both signs and symptoms of dry eye disease in diabetic patients with all DED subtypes and severity categories.

## 1. Introduction

The tear film is a complex structure composed of a mucoaqueous layer and a surface lipid layer [1] and plays a crucial role in protecting and lubricating the ocular surface [2]. A dysfunction in any of its layers can cause dry eye disease (DED), a multifactorial condition of the ocular surface that represents a significant challenge due to its complex etiology. DED has become a public health concern driven by factors such as the widespread use of technology, population aging, exposure to adverse environmental factors [3], and systemic diseases like type-2 diabetes mellitus (T2DM) [4].

Regarding the association of diabetes with dry eye, available evidence shows that T2DM is associated with all subtypes of DED. Peripheral diabetic neuropathy can compromise corneal innervation, reducing sensitivity and the reflex response of tear production, inducing aqueous-deficient dry eye (ADDE) [4]. Chronic inflammation associated with diabetes contributes to Meibomian gland dysfunction (MGD), reducing tear breakup time and exacerbating evaporative dry eye (EDE) [5]. Furthermore, diabetes mellitus can induce metabolic and microvascular changes that directly affect the ocular surface [6], promoting the accumulation of advanced glycation end products (AGEs) that alter the stability of the tear film [7].

Considering the underlying pathophysiological mechanisms that contribute to the development of DED in patients with T2DM, it could be hypothesized that the use of an artificial tear that acts on all the layers of the tear film would represent a suitable intervention to restore ocular homeostasis in diabetic patients. A propylene glycol-hydroxypropyl guar nanoemulsion has been shown in previous studies to improve tear film stability in nondiabetic patients with all types of DED [8,9,10]; however, since signs and symptoms of DED may be more severe in diabetic than in non-diabetic patients, it is relevant to evaluate the performance of this nanoemulsion in patients with T2DM.

## 2. Materials and Methods

This was a single-center, open-label, single-arm, interventional study of adult patients with type-2 diabetes mellitus and DED of all subtypes (aqueous-deficient, evaporative, and mixed) that adhered to the latest tenets of the Declaration of Helsinki.

All patients 18 years or older with a history >3 years of T2DM who attended the dry eye clinic were screened to confirm DED and classify the dry eye subtype:

(a) A tear meniscus height (TMH) < 0.20 mm indicative of a low tear volume, i.e., ADDE [11];
(b) A Non-Invasive Keratograph Break-up Time (NIKBUT) < 5 s, indicative of evaporative dry eye. The meibomian gland expressibility score (MGES) was recorded on a 0 to 3 scale as follows: 0 = all glands expressible; 1 = 3–4 glands expressible; 2 = 1–2 glands expressible; 3 = no glands expressible (modified from Petricek et al. [12]), as well as the infrared meibography score, both indicative of meibomian gland disease (MGD) per the DEWS II diagnostic classification tests for dry eye subtype [11];
(c) If (a) and (b) were met, the case was classified as mixed dry eye. Patients were required to have an Ocular Surface Disease Index (OSDI) score > 13 to qualify for the study.

The exclusion criteria were the following:

- History of hypersensitivity to the study product or any of its excipients;
- History of any eyelid warming therapy initiated ≤12 weeks before screening;
- Patients using glaucoma medication;
- Contact lens wearers;
- History of ocular trauma, infection, or inflammation in the past three months.

All patients who fulfilled DED diagnostic criteria and agreed to participate in the study signed an informed consent form and underwent a two-week washout period of any artificial tear before Visit 0 (baseline). At Visit 0 and Visit 1, we conducted measurements for all study endpoints, as detailed below. Patients were instructed to use the study treatment three times a day, based on a previous study that showed that this formula provided symptom relief for 8 h [8]. The investigational treatment was locally sourced from Alcon Laboratories, which is a propylene glycol-hydroxypropyl guar (PG-HPG) nanoemulsion (Systane^®^ Complete (Alcon, Inc., Fort Worth, TX, USA)). In this formula, PG acts as a demulcent, HPG has gelling properties, and it also includes a lipid component in nano-sized droplets designed to enhance lipid surface coverage. Additionally, it incorporates a distinctive meshwork technology that functions as a protective barrier for the ocular surface [8].

The primary endpoint was the change of OSDI score from baseline to days 14 and 28. The OSDI questionnaire is a patient-reported outcome measure that evaluates dry eye symptoms and their impact on daily activities. It classifies patients as having mild (score of 13–22), moderate (score of 23–32), or severe (score > 33) dry eye [11]. The secondary endpoint was the NIKBUT change from baseline to days 14 and 28. The NIKBUT is measured using the Keratograph 5 M (K5M, Oculus, Wetzlar, Germany), which projects rings onto the cornea to observe tear film disruption without the use of a fluorescein dye, measuring the time it takes for the appearance of the first break in the tear film after a blink. A shorter NIKBUT suggests a less stable tear film, with expected values in subjects without dry eye of >10 s.

**Figure 1:**
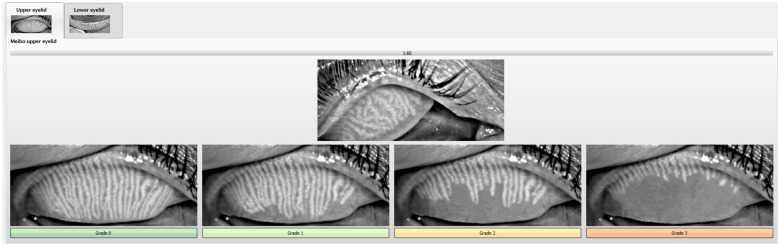
MGD classification using the JENVIS Grading Scale that provides a standardized framework wherein the operator overlays the patient’s eyelid image (in the image, the upper eyelid) onto a series of reference images corresponding to Grades 0–3 of MGD. A sliding bar facilitates direct visual comparison between the patient’s image and the reference images, enabling the operator to determine the closest match in terms of glandular morphology, where the scale generates a numerical value representing the degree of MGD. Although this method is inherently subjective due to its reliance on operator interpretation, it offers a reproducible and semi-quantitative measure that supports imaging-based documentation of disease diagnosis and progression. This image was taken from the Jenvis software of the Keratograph 5 M.

Finally, exploratory endpoints were infrared meibography score, TMH, Redness score, MGES, and Corneal staining scale, all measured with the K5M. Infrared meibography was performed everting the superior and inferior eyelids to capture images used to grade the proportion of MGD with the JENVIS Grading Scale, which is a four-point grading scale that allows easy classification of MGD (see Figure 1). To maintain a consistent eyelid eversion between patients and obtain adequate images for the diagnosis, we used the Meivertor^TM^ (Meivertor, Toronto, ON, Canada), an eyelid eversion tool specifically designed to be used with meibography systems.

The TMH measures the height of the tear film at the lower lid margin. The average values in healthy subjects are >0.20 mm, considered an adequate surrogate for Schirmer’s test [13]. A reduced TMH suggests a deficiency in aqueous tear production, allowing a diagnosis of aqueous-deficient dry eye. The automated redness score evaluates the level of conjunctival hyperemia, an indirect indicator of inflammation. The JENVIS scale grades redness automatically, with higher scores indicating more severe inflammation. The MGES quantifies the volume of secretions expressed from the meibomian glands, with a lower secretion score being indicative of MGD. It is important to note that while meibomian gland scores, encompassing both quantity and quality of secretions, are frequently utilized in dry eye research, their assessment remains inherently subjective. For instance, interobserver variability may lead to discrepancies, where one investigator might classify a secretion as scarce, while another might categorize the same secretion as “moderate”. Additionally, the qualitative assessment of meibum is based on its physical characteristics: clear meibum is typically classified as normal meibum, whereas meibum with a toothpaste-like consistency is deemed poor quality meibum. Given the subjective nature of these evaluations, we employed a modified version of a widely recognized scale [12] to focus only on the approximate volume of meibum expressed, acknowledging that this endpoint remains exploratory due to its non-quantitative and observer-dependent nature. Finally, the K5M software includes the JENVIS corneal staining grading scale, which facilitates the evaluation of fluorescein corneal staining patterns. Fluorescein dye is a diagnostic tool that selectively binds to areas of corneal epithelial disruption. Under blue light illumination, the dye fluoresces, highlighting corneal epithelial irregularities, as typically observed in cases of keratoconjunctivitis sicca. The patient’s images are compared with standardized reference images displayed on-screen, and the software assigns a grading score based on the intensity and distribution of the staining patterns. Higher scores indicate more extensive corneal epithelial damage, reflecting greater severity of dry eye disease.

### Statistical Analysis

Data were collected in an Excel spreadsheet (Microsoft Office Excel, Microsoft, Redmond, WA, USA) without identifying data and transferred to an SPSS file (SPSS Statistics Version 25.0, IBM, Armonk, NY, USA) for statistical analysis. The numerical variables were described with central tendency and dispersion measures (mean ± standard deviation (SD)) and the categorical variables with absolute numbers and percentages. The relationship between categorical variables was analyzed using the chi-square test for unrelated variables and the McNemar’s test for correlated variables. The mean difference between numerical variables was analyzed with the Student’s *t*-test (for independent or related samples). A *p*-value < 0.05 was considered statistically significant. A sample size of 80 eyes was calculated for the primary endpoint as recommended by the TFOS DEWS II Diagnostic Methodology report [11], considering a 10% dropout.

## 3. Results

We included 86 eyes that met the inclusion and none of the exclusion criteria. The mean age of the patients was 58.91 years, with an SD of 12.29 years. There was a predominance of females (69.8% female vs. 30.2% male) with a male:female ratio of 1.00:2.30. The mean time of diagnosis of diabetes mellitus was 10.26 years, with an SD of 7.15.

**Figure 2:**
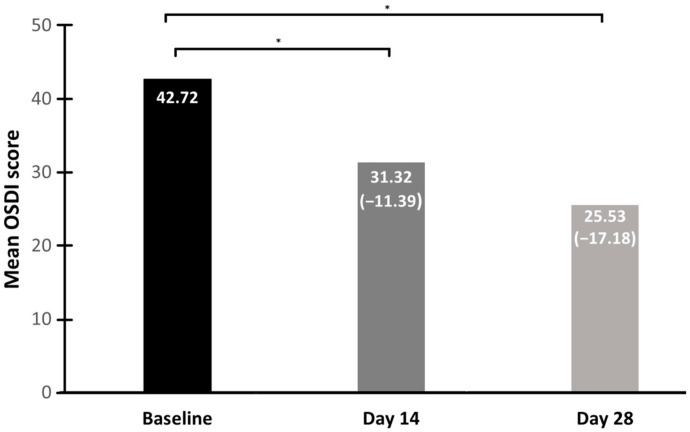
Mean OSDI score changes between baseline and Days 14 and 28. * *p* < 0.001 vs. baseline.

**Figure 3:**
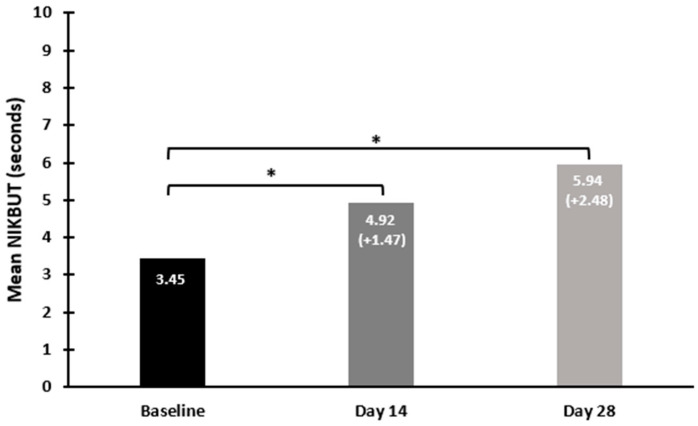
Mean NIKBUT changes from baseline to Days 14 and 28. * *p* < 0.001 vs. baseline.

Table 1 shows the results for the study endpoints: the change in OSDI score from baseline to Days 14 and 28, the change in NIKBUT from baseline to Days 14 and 28, and the change from baseline to Day 28 for redness score and TMH. There were statistically significant decreases in the OSDI score from baseline to Days 14 and 28 and the redness score between baseline and Day 28. Likewise, there were statistically significant increases in the NIKBUT between baseline and Days 14 and 28. For the TMH, there were no statistically significant changes at the two time points evaluated. Figure 2 and Figure 3 show the changes in OSDI and NIKBUT scores at Days 14 and 28.

It was reported that to establish a minimal clinically important difference (MCID), the decrease in the OSDI score should range from 4.5 to 7.3 for mild to moderate DED and from 7.3 to 13.4 for severe DED [14]. Figure 2 depicts the results of the overall population, where the MCID was 11.39 on Day 14 and 17.18 on Day 28.

We also report the change from baseline to Day 28 for the MGES and corneal staining scores. Since their scales represent categories, they are reported as differences between proportions, observing a change in proportion at follow-up, with the highest MGES categories having a greater proportion at visit 28 vs. baseline (*p* = 0.001, Table 2). The change in the proportion of corneal staining was also analyzed (Table 3). Here, it is observed that most eyes changed to lower categories at the end of the follow-up, observing categories 0, 1, and 2 at the final visit. The *p*-value of the McNemar test was not obtained since there were no eyes with severe staining categories (3 and 4) on the last visit.

Since the OSDI questionnaire classifies patients into mild, moderate, and severe dry eye as explained before [11], we performed a sub-analysis to evaluate if there were differences in the results, as well as to assess the MCID for each severity category. The results are shown as Supplementary Materials in Tables S1–S9.

There were 12 eyes in the mild OSDI category, 14 in the moderate OSDI category, and 60 in the severe OSDI category. For eyes with mild and moderate OSDI, the results were the same as for the analysis of the entire population, observing statistically significant differences in the OSDI score, NIKBUT, redness score, and corneal staining. The variables infrared meibography and TMH did not have statistically significant changes. The only endpoint that showed a difference compared to the results of the whole population was the change in the proportion of MGES since no statistically significant differences were observed. Regarding the MCID, this was 9.33 and 8.43 on Day 14 and 9.83 and 14.43 on Day 28 for eyes with mild and moderate categories, respectively.

Regarding eyes with OSDI classified as severe, the results were the same as those of the analysis of all patients, including the MGES result; that is, there was a greater proportion of the lower MGES categories at the follow-up visit (Table 1). The MCID in the severe OSDI category was 12.5 on Day 14 and 19.3 on Day 28.

Finally, we evaluated whether patients with an aqueous deficiency component differed in the OSDI and NIKBUT results compared to the overall population of the study. For this purpose, we performed a sub-analysis of patients who had a TMH < 0.20 mm to consider aqueous-deficient dry eye (3), observing that the OSDI value was 47.48 + 18.12 at baseline, 41.74 + 19.29 on Day 14 and 38.59 + 18.54 on Day 28, with a *p*-value < 0.001 for the comparison between baseline and both visits. The MCID was 5.74 for Day 14 and 8.89 for Day 28. For the NIKBUT, the values were 3.47 + 1.14 s at baseline, 4.77 + 1.71 s at Day 14, and 6.078 + 1.61 s at Day 28, with a *p*-value < 0.001 for the comparison between baseline and both visits.

All measurements contained in this table were taken using the Keratograph 5 M (K5M, Oculus, Wetzlar, Germany).

## 4. Discussion

Dry eye disease has become increasingly prevalent due to environmental factors such as air pollution and prolonged exposure to digital screens. In individuals without systemic comorbidities, the body’s inherent homeostatic mechanisms are more effective in compensating for tear film instability. However, in patients with underlying systemic conditions such as diabetes mellitus, the management of DED becomes more complex due to the interplay of metabolic dysregulation and ocular surface pathology. Consequently, evaluating the available treatments for efficacy in this patient population is essential, as their response to treatment may substantially differ from that observed in systemically healthy individuals.

The population of our study reflects patients with a duration of about ten years of T2DM and with all subtypes of DED. The selected primary endpoint was the OSDI score since the patient’s symptoms should be the leading guide to evaluate the efficacy of a treatment, especially considering that dry eye can have symptoms without signs [15]. The OSDI questionnaire evaluates three subscales related to eye discomfort, functionality, and environmental factors, so it comprehensively addresses the impact of DED on everyday life. Our results showed a statistically significant decrease in the OSDI score, measured two and four weeks after treatment initiation (baseline), reflecting a relevant improvement that persisted at least for a month. As a secondary endpoint, we evaluated the NIKBUT, a marker of tear film instability, observing statistically significant increases at the two visits evaluated. The results of the primary and secondary endpoints demonstrate improvement not only of the symptoms perceived by the patient in all areas impacted by DED but also in the objective signs documented in the examination and by the K5M. These results suggest that this formula is as effective in diabetic patients as it is in systemically healthy subjects, as previously reported [8,16,17].

However, we wanted to explore additional DED-related parameters in this specific patient population four weeks after treatment initiation. For example, the bulbar redness score automatically provided by the K5M showed a statistically significant decrease one month after treatment initiation, which is unsurprising considering that bulbar redness is an indirect reflection of inflammation; if the tear film is more stable, consequently there will be less inflammation [18], and therefore the more severe bulbar redness. Consequently, the observed increase in NIKBUT, i.e., a more stable tear film, is accompanied by a decrease in the bulbar redness score. Also, the positive corneal staining with fluorescein reflects the presence of corneal epithelial defects, that is, the existence of significant instability of the tear film with inflammatory markers that induce corneal barrier disruption due to lysis of epithelial tight junctions [18]. We observed that one month after starting treatment, the proportion of eyes with corneal staining decreased significantly, to the extent that there were no eyes in the most severe categories at the final visit. This possibly reflects that the study treatment improved ocular surface homeostasis, allowing proper regeneration of the corneal epithelium with the presence of a more stable tear film.

An intriguing finding was the increased proportion of higher MGES categories at the final visit, indicative of worse MG expressibility. However, this outcome can be explained by the study design, specifically, an essential part of DED treatment involves eyelid warming therapies, which include at-home warm masks or compresses to thin the meibum, as well as in-office devices with various mechanisms aimed at improving MG function. To ensure study control, patients were instructed to avoid both at-home and in-office warming therapies four weeks prior to screening. We believe this contributed to the observed decline in MG expressibility at the final visit across the general population. Despite this, the reduction in MG expressibility did not outweigh the overall improvements in DED signs and symptoms. This result highlights the importance of incorporating both pharmacological therapies and warming treatments in the management of DED.

Infrared meibography and TMH were the only endpoints that remained unchanged following treatment. This outcome is expected, as the formulation used in the study is primarily designed to protect the ocular surface through its polymer-based properties, enhance hydration via demulcents, and stabilize the tear film with its lipid components, rather than to significantly increase tear volume. Nevertheless, we conducted a sub-analysis on patients with a TMH < 0.20 mm, a threshold indicative of aqueous deficiency, to determine whether primary and secondary endpoint results diverged from those of the general population. In this subgroup, we observed statistically significant improvements in both the OSDI score and NIKBUT, demonstrating that the absence of changes in tear film volume did not preclude improvements in tear film quality.

Lastly, regarding infrared meibography, we did not anticipate any changes following the use of the study treatment, as, to the best of our knowledge, no pharmacological or device-based interventions have been shown to reverse MG atrophy [19].

## 5. Conclusions

The results of this study underscore the efficacy of the study formula in significantly improving both signs and symptoms of DED across all subtypes in patients with T2DM. The observed improvements in tear film stability parameters show that the treatment addresses the complex pathophysiology of DED in this high-risk population that often presents more severe and complex ocular surface disease. Further research is warranted to explore long-term benefits and potential synergistic effects with other DED therapies.

## Figures and Tables

**Table 1:** Mean differences at baseline and subsequent visits.

Outcome	Mean ± SD	*p*-Value
Baseline OSDI score	42.72 ± 17.69	
OSDI (Day 14)	31.33 ± 18.01	˂0.001
OSDI (Day 28)	25.53 ± 17.14	˂0.001
Baseline NIKBUT (seconds)	3.45 ± 1.17	
NIKBUT (Day 14)	4.92 ± 1.6	˂0.001
NIKBUT (Day 28)	5.94 ± 1.48	˂0.001
Baseline infrared meibography score	1.48 ± 0.93	
Infrared meibography (Day 28)	1.47 ± 0.92	0.279
Baseline TMH (mm)	0.25 ± 0.10	
TMH (Day 28)	0.25 ± 0.08	0.086
Baseline redness score	1.88 ± 0.68	
Redness score (Day 28)	1.40 ± 0.59	˂0.001

**Table 2:** Proportion difference in meibomian gland expressibility score (MGES).

			MGES (Day 28)				Total
			0	1	2	3	
MG expressibility score	0	Count	8	8	0	0	16
		% of the total	9.3%	9.3%	0.0%	0.0%	18.6%
	1	Count	0	18	5	0	23
		% of the total	0.0%	20.9%	5.8%	0.0%	26.7%
	2	Count	0	1	18	0	19
		% of the total	0.0%	1.2%	20.9%	0.0%	22.1%
	3	Count	0	0	6	22	28
		% of the total	0.0%	0.0%	7.0%	25.6%	32.6%
Total		Count	8	27	29	22	86
		% of the total	9.3%	31.4%	33.7%	25.6%	100.0%
*p* = 0.001							

Meibomian gland expressibility score (MGES) was recorded on a 0 to 3 scale, as explained in Section 2.

**Table 3:** Proportion difference of JENVIS corneal staining scale (CS).

			CS (Day 28)			Total
			0	1	2	
Baseline corneal staining	0	Count	54	0	0	54
		% of the total	62.8%	0.0%	0.0%	62.8%
	1	Count	16	2	0	18
		% of the total	18.6%	2.3%	0.0%	20.9%
	2	Count	0	10	0	10
		% of the total	0.0%	11.6%	0.0%	11.6%
	3	Count	0	3	0	3
		% of the total	0.0%	3.5%	0.0%	3.5%
	4	Count	0	0	1	1
		% of the total	0.0%	0.0%	1.2%	1.2%
Total		Count	70	15	1	86
		% of the total	81.4%	17.4%	1.2%	100.0%

*p*-value with the Mcnemar test cannot be calculated since categories 3 and 4 were absent at follow-up. Corneal staining was measured using the JENVIS corneal staining grading scale included in the Keratograph 5 M (K5M, Oculus, Wetzlar, Germany).

## Data Availability

The original contributions presented in this study are included in the article/Supplementary Materials. Further inquiries can be directed to the corresponding author(s).

## Supplementary Materials

The following supporting information can be downloaded at: https://www.mdpi.com/article/10.3390/biomedicines13040763/s1, Table S1: Mean differences at baseline vs. subsequent visits in the 12 eyes with mild OSDI. Table S2: MGES proportion comparison: mild OSDI. Table S3: Corneal staining proportion comparison: mild OSDI. Table S4: Mean differences at baseline vs. subsequent visits in the 14 eyes with moderate OSDI. Table S5: MGES proportion comparison: moderate OSDI. Table S6: Corneal staining proportion comparison: moderate OSDI. Table S7: Mean differences at baseline vs. subsequent visits in the 60 eyes with severe OSDI. Table S8: MGES proportion comparison: severe OSDI. Table S9: Corneal staining proportion comparison: severe OSDI.
