# Supplementary material for: Effect of a Nano-Sized Lipid-Based Eye Drop on Diabetic Dry Eye
Source: Biomedicines. 2025 Mar 21;13(4):763. doi: 10.3390/biomedicines13040763 (PMC12024559; doi:10.3390/biomedicines13040763)
Supplement: Supplementary file 1 [file biomedicines-13-00763-s001.zip › biomedicines-3520732-supplementary.pdf]

Supplementary Data

Table S1. Mean differences at baseline vs subsequent visits in the 12 eyes with mild OSDI.

Outcome	Mean ± SD	p-value
Baseline OSDI score	17.33 ± 2.67	
OSDI (Day 14)	8.00 ± 3.81	<0.001
Baseline OSDI score	17.33 ± 2.67	
OSDI (Day 28)	7.50 ± 2.39	<0.001
Baseline NIKBUT (seconds)	3.26 ± 1.24	
NIKBUT (Day 14)	5.59 ± 2.06	<0.001
Baseline NIKBUT (seconds)	3.26 ± 1.24	
NIKBUT (Day 28)	5.96 ± 1.45	<0.001
Baseline infrared meibography (score)	1.60 ± 1.08	
Infrared meibography (Day 28)	1.61 ± 1.09	0.767
Baseline TMH (mm)	0.29 ± 0.09	
TMH (Day 28)	0.28 ± 0.07	0.210
Baseline redness score	2.00 ± 0.90	
Redness score (Day 28)	1.51 ± 0.90	0.002

Table S2. MGES proportion comparison: mild OSDI.

			MGES (Day 28)				Total
			0	1	2	3	
MG expressibility score	0	Count	3	1	0	0	4
		% of the total	25.0%	8.3%	0.0%	0.0%	33.3%
	1	Count	0	2	0	0	2
		% of the total	0.0%	16.7%	0.0%	0.0%	16.7%
	2	Count	0	0	2	0	2
		% of the total	0.0%	0.0%	16.7%	0.0%	16.7%
	3	Count	0	0	0	4	4
		% of the total	0.0%	0.0%	0.0%	33.3%	33.3%
Total		Count	3	3	2	4	12
		% of the total	25.0%	25.0%	16.7%	33.3%	100.0%
		P= 0.317					

https://doi.org/10.3390/xxxxx

Table S3. Corneal staining proportion comparison: mild OSDI

			CS (Day 28)		Total
			0	1	
Baseline corneal staining	0	Count	6	0	6
		% of the total	50.0%	0.0%	50.0%
	1	Count	4	0	4
		% of the total	33.3%	0.0%	33.3%
	2	Count	0	2	2
		% of the total	0.0%	16.7%	16.7%
Total		Count	10	2	12
		% of the total	83.3%	16.7%	100.0%

P= The p-value with the Mcnemar test cannot be calculated since categories 3 and 4 were absent at follow-up.

Table S4. Mean differences at baseline vs subsequent visits in the 14 eyes with moderate OSDI.

**Outcome**	**Mean ± SD**	**p-value**
Baseline OSDI score	26.00 ± 2.60	
OSDI (Day 14)	17.57 ± 5.37	<0.001
Baseline OSDI score	26.00 ± 2.60	
OSDI (Day 28)	11.57 ± 4.10	<0.001
Baseline NIKBUT (seconds)	3.72 ± 1.34	
NIKBUT (Day 14)	4.43 ± 0.63	0.043
Baseline NIKBUT (seconds)	3.72 ± 1.34	
NIKBUT (Day 28)	6.57 ± 1.19	<0.001
Baseline infrared meibography (score)	0.96 ± 0.76	
Infrared meibography (Day 28)	0.97 ± 0.76	0.865
Baseline TMH (mm)	0.30 ± 0.14	
TMH (Day 28)	0.30 ± 0.13	0.678
Baseline redness score	1.90 ± 0.57	
Redness score (Day 28)	1.36 ± 0.51	<0.001

Table S5. MGES proportion comparison: moderate OSDI.

			MGES (Day 28)			Total
			1	2	3	
MG expressibility score	1	Count	3	0	0	3
		% of the total	21.4%	0.0%	0.0%	21.4%
	2	Count	1	2	0	3

		% of the total	7.1%	14.3%	0.0%	21.4%
	3	Count	0	2	6	8
		% of the total	0.0%	14.3%	42.9%	57.1%
Total		Count	4	4	6	14
		% of the total	28.6%	28.6%	42.9%	100.0%
		P= 0.223				

Table S6. Corneal staining proportion comparison: moderate OSDI.

			CS (Day 28)	
			0	Total
Baseline corneal staining	0	Count	12	12
		% of the total	85.7%	85.7%
	1	Count	2	2
		% of the total	14.3%	14.3%
Total		Count	14	14
		% of the total	100.0%	100.0%

P= The p-value with the Mcnemar test cannot be calculated since categories 3 and 4 were absent at follow-up.

Table S7. Mean differences at baseline vs subsequent visits in the 60 eyes with severe OSDI.

**Outcome**	**Mean ± SD**	**p-value**
Baseline OSDI	51.70 ± 12.99	
OSDI (Day 14)	39.20 ± 15.48	<0.001
Baseline OSDI	51.70 ± 12.99	
OSDI (Day 28)	32.40 ± 16.07	<0.001
Baseline NIKBUT	3.43 ± 1.12	
NIKBUT (Day 14)	4.90 ± 1.64	<0.001
Baseline NIKBUT	3.43 ± 1.12	
NIKBUT (Day 28)	5.79 ± 1.53	<0.001
Baseline infrared meibography	1.58 ± 0.91	
Infrared meibography (Day 28)	1.55 ± 0.90	.209
Baseline TMH mm	0.23 ± 0.08	
TMH (Day 28) mm	0.23 ± 0.07	.224
Baseline redness score	1.85 ± 0.67	
Redness score 28 days	1.38 ± 0.54	<0.001

Table S8. MGES proportion comparison: severe OSDI.

			MGES (Day 28)				Total
			0	1	2	3	
MG expressibility score	0	Count	5	7	0	0	12
		% of the total	8.3%	11.7%	0.0%	0.0%	20.0%
	1	Count	0	13	5	0	18
		% of the total	0.0%	21.7%	8.3%	0.0%	30.0%
	2	Count	0	0	14	0	14
		% of the total	0.0%	0.0%	23.3%	0.0%	23.3%
	3	Count	0	0	4	12	16
		% of the total	0.0%	0.0%	6.7%	20.0%	26.7%
Total		Count	5	20	23	12	60
		% of the total	8.3%	33.3%	38.3%	20.0%	100.0%
P= 0.001							

Table S9. Corneal staining proportion comparison: severe OSDI.

			CS (Day 28)			Total
			0	1	2	
Baseline corneal staining	0	Count	36	0	0	36
		% of the total	60.0%	0.0%	0.0%	60.0%
	1	Count	10	2	0	12
		% of the total	16.7%	3.3%	0.0%	20.0%
	2	Count	0	8	0	8
		% of the total	0.0%	13.3%	0.0%	13.3%
	3	Count	0	3	0	3
		% of the total	0.0%	5.0%	0.0%	5.0%
	4	Count	0	0	1	1
		% of the total	0.0%	0.0%	1.7%	1.7%
Total		Count	46	13	1	60
		% of the total	76.7%	21.7%	1.7%	100.0%

P= The p-value with the Mcnemar test cannot be calculated since categories 3 and 4 were absent at follow-up.
